# Supplementary material for: The Use of Flocked Swabs with a Protective Medium Increases the Recovery of Live Brucella spp. and DNA Detection
Source: Microbiol Spectr. 2021 Nov 17;9(3):e00728-21. doi: 10.1128/Spectrum.00728-21 (PMC8597647; doi:10.1128/Spectrum.00728-21)
Supplement: SUPPLEMENTAL FILE 1 — Supplemental material. Download SPECTRUM00728-21_Supp_1_seq8.pdf, PDF file, 1.4 MB [file spectrum00728-21_supp_1_seq8.pdf]

**Supplemental material**

**Figure. S1.** Control of efficacy of DNA extraction and qPCR inhibition by internal process control (IPC) Ct levels in each individual repeat. The total DNA extracted from all treated swabs types (run positive control= R, positive control = PC, viscose = S1, cotton-wool = S2, polyester = S3, nylon flocked = S4, nylon flocked with medium = S5, Ca-alginate = S6 and polyester flocked = S7) were tested individually from spiked swabs. The red squares present the limits of acceptability – three Ct values above and below the R of each run separately. There was no qPCR inhibition when compared from the swabs spiked with A) *B. melitensis* 16M strain; B) *B. abortus* 544 strain; C) *B. suis* Thomsen strain.

**Table. S1.** Comparison of *Brucella* direct detection performance from seven tested swab types and positive control. After ANOVA was performed for each storage condition separately, a post hock T-test analysis was used to compare individual effect of each swab on classical bacteriology and molecular detection. The results show the different significance levels of each swab compared to positive control for three used strains in each storage condition. The effect of each analysis is presented as p-value and 95% Confidence Interval (CI). NS – not significant.

**Table. S2.** Comparison of bacteriology and qPCR recovery rates based on different *Brucella* strains, storage conditions and swab types. The mean, minimal and maximal values of bacterial and DNA recovery rates are presented regarding the *Brucella* strains, storage conditions, and swab type used. The results show significant variations in recovery rates of live bacteria between various swabs, based on different *Brucella* strains and storage conditions. In the same time, the variations in detectability of *Brucella* DNA by qPCR are significantly lower than live bacteria recovery as three values (mean, min, max) are compared.

# Fig. S1

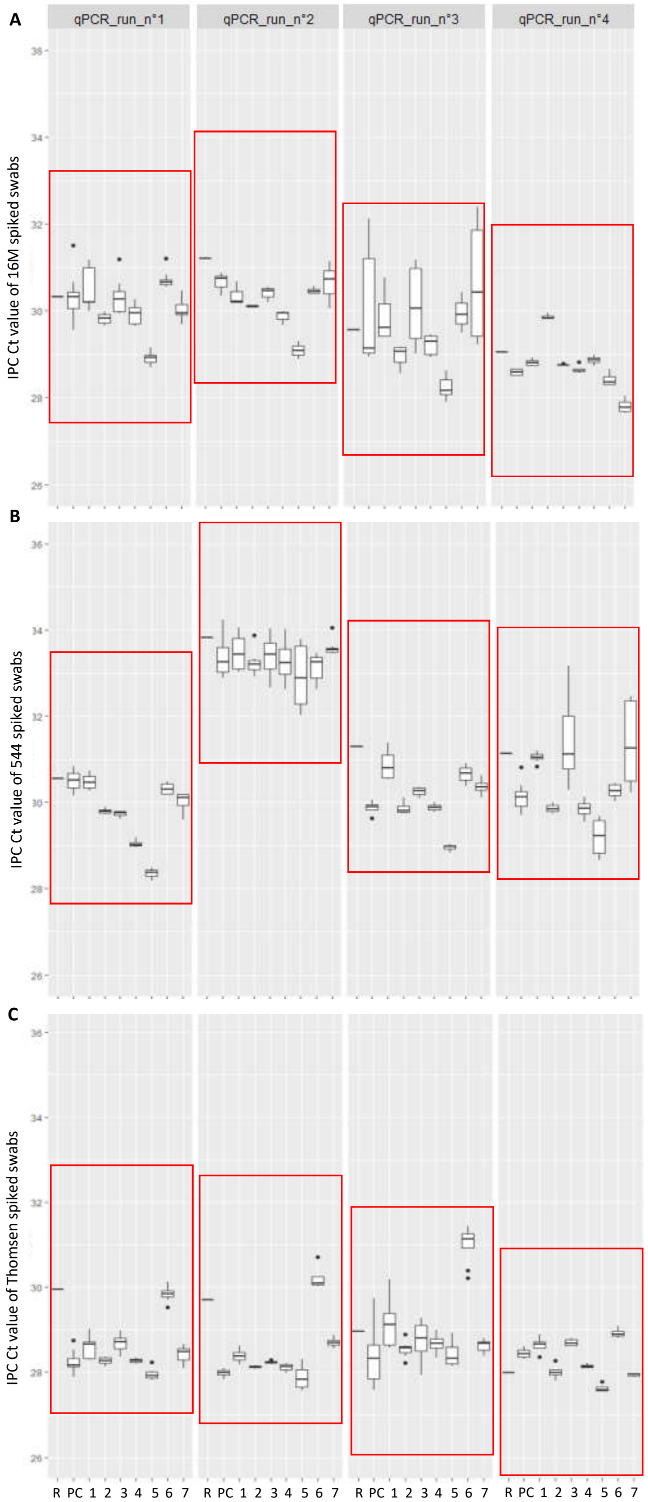
A
B
C
qPCR_run_n°1
qPCR_run_n°2
qPCR_run_n°3
qPCR_run_n°4
IPC Ct value of 16M spiked swabs
IPC Ct value of 544 spiked swabs
IPC Ct value of Thomsen spiked swabs
36
34
32
30
28
26
R PC 1 2 3 4 5 6 7

# Table S1

| Brucella strains | Storage condition | Recovery type | Post-Hoc Student T-test analysis (Tukey's test) | Swab Type compared to CP (2 ml of spiked PBS) | | | | | | |
|---|---|---|---|---|---|---|---|---|---|---|
| | | | | S1 | S2 | S3 | S4 | S5 | S6 | S7 |
| Three strains (16M, 554 and Thomsen) | DT | Bacteriology | difference (95% CI) | -20.0 (-36.1 / -3.8) | -64.1 (-80.2 / -47.9) | NS | NS | NS | -42.8 (-58.9 / -26.6) | -30.3 (-46.5 / -14.2) |
| | | | adjusted *p*- value | 0.005 | <0.0001 | 1.0 | 1.0 | 0.25 | <0.0001 | <0.0001 |
| | | qPCR | difference (95% CI) | -11.8 (-15.9 / -7.7) | -7.4 (-11.5 / -3.3) | -4.8 (-8.9 / -0.7) | NS | 18.9 (14.8 / 23.0) | -17.9 (-22.0 / -13.8) | NS |
| | | | adjusted *p-value* | <0.0001 | <0.0001 | 0.0089 | 0.57 | <0.0001 | <0.0001 | 0.15 |
| | 72h_+4°C | Bacteriology | difference (95% CI) | -52.7 (-64.2 / -41.3) | -81.5 (-92.9 /-70.1) | -32.7 (-44.1 / -21.3) | NS | 15.7 (4.3 / 27.1) | -81.5 (-92.9 / -70.1) | -33.3 (-44.7 / -21.8) |
| | | | adjusted *p*- value | <0.0001 | <0.0001 | <0.0001 | 1.0 | 0.001 | <0.0001 | <0.0001 |
| | | qPCR | difference (95% CI) | -8.6 (-12.8 / -4.4) | NS | -4.7 (-8.8 / -0.6) | NS | 27.3 (23.2 / 31.4) | -6.1 (-10.2 / -1.9) | -5.5 (-9.6 / -1.4) |
| | | | adjusted *p*- value | <0.0001 | 1.0 | 0.014 | 1.0 | <0.0001 | 0.0003 | 0.0015 |
| | 72h_-20°C | Bacteriology | difference (95% CI) | -14.8 (-30.5 / 0.9) | -36.5 (-52.1 / -21.0) | -44.8 (-60.3 / -29.3) | NS | 35.1 (19.4 / 50.5) | -36.8 (-52.3 / -21.3) | -39.1 (-54.6 / -23.6) |
| | | | adjusted *p*- value | 0.079 | <0.0001 | <0.0001 | 0.63 | <0.0001 | <0.0001 | <0.0001 |
| | | qPCR | difference (95% CI) | -6.1 (-11.5 /-0.5) | NS | NS | NS | 8.7 (3.2 / 14.2) | NS | NS |
| | | | adjusted *p*- value | 0.022 | 0.052 | 0.091 | 1.0 | <0.0001 | 0.69 | 0.98 |
| 16M | DT | Bacteriology | difference (95% CI) | -18.3 (-35.7 / -0.9) | -32.8 (-50.2 / -15.4) | NS | NS | 11.1 (-6.3 / 28.5) | -24.9 (-42.3 / -7.5) | -43.3 (-60.6 / -25.9) |
| | | | adjusted *p*- value | 0.032 | <0.0001 | 1.0 | 1.0 | 0.48 | 0.0008 | <0.0001 |
| | | qPCR | difference (95% CI) | -9.2 (-18.1 / -0.3) | NS | -5.6 (-14.5 / 3.3) | NS | 19.3 (10.3 / 28.2) | -17.1 (-26.0 / -8.2) | NS |
| | | | adjusted *p*- value | 0.039 | 0.62 | 0.50 | 0.84 | <0.0001 | <0.0001 | 0.95 |
| | 72h_+4°C | Bacteriology | difference (95% CI) | -55.2 (-74.4 / -36.0) | -83.6 (-102.8 / -64.5) | NS | NS | NS | -83.6 (-102.8 / -64.5) | -44.1 (-63.2 / -24.9) |
| | | | adjusted *p*- value | <0.0001 | <0.0001 | 0.08 | 1.0 | 0.13 | <0.0001 | <0.0001 |
| | | qPCR | difference (95% CI) | -7.8 (-13.9 / -1.7) | NS | NS | NS | 30.5 (24.6 / 36.4) | -7.3 (-13.3 / -1.4) | NS |
| | | | adjusted *p*- value | 0.004 | 1.0 | 0.07 | 1.0 | <0.0001 | 0.006 | 0.06 |
| | 72h_-20°C | Bacteriology | difference (95% CI) | NS | -38.0 (-60.9 / -15.1) | -62.8 (-85.7 / -39.9) | NS | 23.8 (0.9 / 46.6) | -65.7 (-88.6 / -42.8) | -45.6 (-68.5 / -22.7) |
| | | | adjusted *p*- value | 0.45 | <0.0001 | <0.0001 | 1.0 | 0.04 | <0.0001 | <0.0001 |
| | | qPCR | difference (95% CI) | NS | NS | NS | NS | NS | NS | NS |
| | | | adjusted *p*- value | 0.77 | 0.31 | 0.98 | 1.0 | 0.25 | 1.0 | 0.21 |

# Table S1

| | | | | | | | | | | |
|---|---|---|---|---|---|---|---|---|---|---|
| 544 | DT | Bacteriology | difference (95% CI) | -20.7 (-32.0 / -9.5) | -82.7 (-93.9 / -71.4) | NS | NS | NS | -26.7 (-37.9 / -15.5) | -17.7 (-28.9 / -6.5) |
| | | | adjusted *p*- value | <0.0001 | <0.0001 | 1.0 | 1.0 | 0.26 | <0.0001 | 0.0002 |
| | | qPCR | difference (95% CI) | -16.5 (-22.0 / -11.0) | -7.0 (-12.6 / -1.5) | NS | NS | 22.4 (16.8 / 27.9) | -15.1 (-20.6 / -9.6) | NS |
| | | | adjusted *p*- value | <0.0001 | 0.004 | 0.18 | 1.0 | <0.0001 | <0.0001 | 0.57 |
| | 72h_+4°C | Bacteriology | difference (95% CI) | -51.9 (-61.6 / -42.2) | -89.8 (-99.5 / -80.0) | -33.5 (-43.3 / -23.8) | NS | NS | -89.8 (-99.5 / -80.0) | -37.5 (-47.2 / -27.7) |
| | | | adjusted *p*- value | <0.0001 | <0.0001 | <0.0001 | 0.50 | 0.11 | <0.0001 | <0.0001 |
| | | qPCR | difference (95% CI) | -6.5 (-12.9 / -0.2) | NS | NS | NS | 27.3 (20.9 / 33.7) | NS | -6.4 (-12.8 / -0.1) |
| | | | adjusted *p*- value | 0.04 | 1.0 | 1.0 | 0.97 | <0.0001 | 1.0 | 0.047 |
| | 72h_-20°C | Bacteriology | difference (95% CI) | NS | -34.1 (-47.8 / -20.3) | -34.1 (-47.8 / -20.3) | NS | 34.9 (21.2 / 48.6) | NS | -34.1 (-47.8 / -20.3) |
| | | | adjusted *p*- value | 0.39 | <0.0001 | <0.0001 | 1.0 | <0.0001 | 0.72 | <0.0001 |
| | | qPCR | difference (95% CI) | NS | NS | -8.5 (-15.9 / -1.0) | NS | NS | NS | NS |
| | | | adjusted *p*- value | 0.13 | 0.26 | 0.02 | 0.94 | 0.30 | 1.0 | 0.76 |
| Thomsen | DT | Bacteriology | difference (95% CI) | -20.8 (-36.8 / -4.9) | -76.7 (-92.7 / -60.8) | NS | NS | 18.2 (2.2 / 34.1) | -76.8 (-92.7 / -60.8) | -30.1 (-46.1 / -14.2) |
| | | | adjusted *p*- value | 0.003 | <0.0001 | 1.0 | 1.0 | 0.015 | <0.0001 | <0.0001 |
| | | qPCR | difference (95% CI) | -9.8 (-15.1 / -4.6) | -10.0 (-15.2 / -4.7) | NS | NS | 15.1 (9.8 / 20.3) | -21.5 (-26.8 / -16.3) | NS |
| | | | adjusted *p*- value | <0.0001 | <0.0001 | 0.19 | 0.38 | <0.0001 | <0.0001 | 0.24 |
| | 72h_+4°C | Bacteriology | difference (95% CI) | -51.1 (-69.9 / -32.4) | -71.1 (-89.8 / -52.3) | -46.5 (-65.3 / -27.8) | NS | 21.6 (2.8 / 40.3) | -71.1 (-89.8 / -52.3) | NS |
| | | | adjusted *p*- value | <0.0001 | <0.0001 | <0.0001 | 1.0 | 0.014 | <0.0001 | 0.06 |
| | | qPCR | difference (95% CI) | -11.8 (-16.1 / -7.5) | NS | -8.1 (-12.3 / -3.8) | NS | 24.1 (19.8 / 28.3) | -12.0 (-16.2 / -7.7) | -4.3 (-8.6 / -0.1) |
| | | | adjusted *p*- value | <0.0001 | 0.81 | <0.0001 | 0.20 | <0.0001 | <0.0001 | 0.046 |
| | 72h_-20°C | Bacteriology | difference (95% CI) | -37.6 (-50.8 / -24.4) | -37.6 (-50.8 /-24.4) | -37.6 (-50.8 /-24.4) | -19.5 (-32.7 /-6.4) | 46.2 (33.0 / 59.4) | -37.6 (-50.8 / -24.4) | -37.6 (-50.8 / -24.4) |
| | | | adjusted *p*- value | <0.0001 | <0.0001 | <0.0001 | 0.0005 | <0.0001 | <0.0001 | <0.0001 |
| | | qPCR | difference (95% CI) | -5.4 (-10.3 / -0.5) | NS | NS | NS | 13.7 (8.8 / 18.6) | -7.0 (-11.9 / -2.2) | NS |
| | | | adjusted *p*- value | 0.02 | 0.87 | 0.46 | 0.98 | <0.0001 | 0.0008 | 1.0 |

# Table S2

| *Brucella* strains | Storage condition | Swab Type | Recovery type | Mean recovery value (%) | Min recovery value (%) | Max recovery value (%) |
|---|---|---|---|---|---|---|
| 16M | all | all | Bacteriology | 55.8 | 0 | 105.2 |
| 544 | | | | 50.5 | 0 | 103.8 |
| Thomsen | | | | 37.7 | 0 | 100 |
| 16M | | | qPCR | 67 | 51.8 | 99.3 |
| 544 | | | | 67.3 | 48.5 | 99.3 |
| Thomsen | | | | 69.1 | 49.5 | 101.2 |
| all | DT | all | Bacteriology | 68.2 | 0 | 105.2 |
| | 72h_+4°C | | | 48.2 | 0 | 103.8 |
| | 72h_-20°C | | | 27.5 | 0 | 91.1 |
| | DT | | qPCR | 69 | 48.5 | 99.3 |
| | 72h_+4°C | | | 67.6 | 51.8 | 101.2 |
| | 72h_-20°C | | | 66.7 | 52.8 | 95.1 |
| all | all | PC | Bacteriology | 71.1 | 22.2 | 95.5 |
| | | S1 | | 42.1 | 0 | 76.8 |
| | | S2 | | 10.4 | 0 | 62.9 |
| | | S3 | | 45 | 0 | 91.8 |
| | | S4 | | 68.7 | 0 | 96.2 |
| | | S5 | | 92.2 | 55.7 | 105.2 |
| | | S6 | | 17.4 | 0 | 75.4 |
| | | S7 | | 36.9 | 0 | 74.2 |
| | | PC | qPCR | 69.3 | 55.9 | 78.4 |
| | | S1 | | 60.5 | 48.5 | 69.3 |
| | | S2 | | 64.7 | 52.8 | 73 |
| | | S3 | | 64.4 | 53.5 | 73.5 |
| | | S4 | | 68.6 | 59.1 | 78.8 |
| | | S5 | | 87.6 | 64.3 | 101.2 |
| | | S6 | | 60.3 | 49.4 | 74.8 |
| | | S7 | | 66.8 | 51.8 | 95.1 |
| | | PC | | 86.1 | 61.8 | 95.5 |
| | | S1 | | 66.1 | 35.5 | 76.8 |

| | | | | | | |
|---|---|---|---|---|---|---|
| | | S2 | | 22 | 0 | 62.9 |
| | | S3 | Bacteriology | 85.2 | 68.1 | 91.8 |
| | | S4 | | 88.2 | 70.9 | 96.2 |
| | | S5 | | 98.7 | 89.5 | 105.2 |
| | | S6 | | 43.3 | 0 | 75.4 |
| | | S7 | | 55.8 | 0 | 74.2 |
| | DT | PC | qPCR | 72.6 | 62.5 | 78.4 |
| | | S1 | | 60.8 | 48.5 | 69.3 |
| | | S2 | | 65.3 | 61.2 | 73 |
| | | S3 | | 67.8 | 57.3 | 73.5 |
| | | S4 | | 70.1 | 60.7 | 75.5 |
| | | S5 | | 91.5 | 82.3 | 99.2 |
| | | S6 | | 54.7 | 49.4 | 62.9 |
| | | S7 | | 69.1 | 61.6 | 74.9 |
| all | 72h_+4°C | PC | Bacteriology | 81.5 | 58.4 | 94.5 |
| | | S1 | | 28.7 | 0 | 60.5 |
| | | S2 | | 0 | 0 | 0 |
| | | S3 | | 48.8 | 0 | 75.9 |
| | | S4 | | 81.2 | 62 | 92.6 |
| | | S5 | | 97.2 | 87.9 | 103.8 |
| | | S6 | | 0 | 0 | 0 |
| | | S7 | | 48.2 | 25.2 | 65.6 |
| | | PC | qPCR | 67.5 | 55.9 | 76.7 |
| | | S1 | | 58.8 | 53.1 | 68.4 |
| | | S2 | | 66.6 | 55.3 | 71.7 |
| | | S3 | | 62.8 | 53.5 | 71.1 |
| | | S4 | | 66.8 | 59.1 | 72.8 |
| | | S5 | | 94.7 | 90.9 | 101.2 |
| | | S6 | | 61.4 | 55 | 70.5 |
| | | S7 | | 61.9 | 51.8 | 69.5 |
| | | PC | | 45.8 | 22.2 | 74.6 |
| | | S1 | | 31 | 0 | 60.7 |
| | | S2 | | 9.2 | 0 | 58.3 |

| | | | | | | |
|---|---|---|---|---|---|---|
| | 72h_-20°C | S3 | Bacteriology | 1 | 0 | 23.3 |
| | | S4 | | 36.8 | 0 | 73.7 |
| | | S5 | | 80.7 | 55.8 | 91.1 |
| | | S6 | | 9 | 0 | 35.8 |
| | | S7 | | 6.7 | 0 | 44.7 |
| | | PC | qPCR | 67.7 | 60.9 | 74.9 |
| | | S1 | | 61.7 | 57.5 | 66.2 |
| | | S2 | | 62.2 | 52.8 | 70.7 |
| | | S3 | | 62.6 | 55.7 | 68.7 |
| | | S4 | | 68.8 | 61.9 | 78.8 |
| | | S5 | | 76.5 | 64.3 | 91.8 |
| | | S6 | | 64.7 | 57 | 74.8 |
| | | S7 | | 69.5 | 60.9 | 95.1 |
| | DT | PC | Bacteriology | 90.9 | 86 | 95.5 |
| | | S1 | | 72.6 | 70.2 | 73.7 |
| | | S2 | | 58.2 | 54.5 | 62.9 |
| | | S3 | | 87.8 | 85.1 | 90.2 |
| | | S4 | | 91.8 | 86.5 | 95.9 |
| | | S5 | | 102 | 98.5 | 105.2 |
| | | S6 | | 66 | 60.5 | 71.1 |
| | | S7 | | 47.7 | 0 | 72.3 |
| | | PC | qPCR | 71.8 | 62.5 | 78.4 |
| | | S1 | | 62.6 | 54.7 | 69.1 |
| | | S2 | | 66.7 | 61.2 | 73 |
| | | S3 | | 66.1 | 57.3 | 72.9 |
| | | S4 | | 67.7 | 60.7 | 73.7 |
| | | S5 | | 91 | 85.3 | 98.3 |
| | | S6 | | 54.7 | 52.4 | 56.6 |
| | | S7 | | 68.6 | 61.6 | 74.9 |
| | | PC | Bacteriology | 83.6 | 80.7 | 87.6 |
| | | S1 | | 28.4 | 0 | 60.5 |
| | | S2 | | 0 | 0 | 0 |
| | | S3 | | 65.6 | 51.3 | 75.9 |

| | | | | | | |
|---|---|---|---|---|---|---|
| | | S4 | Bacteriology | 87 | 79.2 | 92.6 |
| | | S5 | | 100.4 | 99.4 | 103 |
| | | S6 | | 0 | 0 | 0 |
| 16M | 72h_+4°C | S7 | | 39.6 | 25.2 | 52.8 |
| | | PC | qPCR | 64 | 55.9 | 71 |
| | | S1 | | 56.2 | 53.8 | 61.1 |
| | | S2 | | 64.8 | 57.5 | 71.1 |
| | | S3 | | 58.4 | 53.5 | 61.5 |
| | | S4 | | 63.4 | 59.1 | 68 |
| | | S5 | | 94.5 | 91.4 | 97.6 |
| | | S6 | | 56.7 | 55 | 57.7 |
| | | S7 | | 58.2 | 51.8 | 62.1 |
| | 72h_-20°C | PC | Bacteriology | 65.7 | 52.4 | 74.6 |
| | | S1 | | 50.7 | 34.7 | 59.3 |
| | | S2 | | 27.7 | 0 | 58.3 |
| | | S3 | | 2.9 | 0 | 23.3 |
| | | S4 | | 60.7 | 48 | 73.7 |
| | | S5 | | 89.5 | 88.3 | 91.1 |
| | | S6 | | 0 | 0 | 0 |
| | | S7 | | 20.1 | 0 | 44.7 |
| | | PC | qPCR | 67.3 | 63.6 | 71.7 |
| | | S1 | | 61.1 | 57.6 | 63.9 |
| | | S2 | | 57.8 | 53.9 | 61.4 |
| | | S3 | | 63.5 | 61.7 | 64.6 |
| | | S4 | | 69.2 | 65.1 | 76 |
| | | S5 | | 76.8 | 64.3 | 91.8 |
| | | S6 | | 66.4 | 57 | 74.8 |
| | | S7 | | 77.2 | 60.1 | 95.1 |
| | | PC | Bacteriology | 90.6 | 83 | 95.5 |
| | | S1 | | 69.9 | 56.8 | 76.8 |
| | | S2 | | 7.9 | 0 | 32.9 |
| | | S3 | | 89 | 86.1 | 91.8 |
| | | S4 | | 93.4 | 91.4 | 96.2 |

| | | | | | | |
|---|---|---|---|---|---|---|
| | | S5 | | 99.1 | 95.4 | 103.6 |
| | | S6 | | 63.9 | 51.2 | 75.4 |
| | DT | S7 | | 72.9 | 70 | 74.3 |
| | | PC | qPCR | 71.3 | 69.8 | 73.6 |
| | | S1 | | 54.9 | 48.5 | 58.6 |
| | | S2 | | 64.3 | 61.8 | 68 |
| | | S3 | | 66.8 | 62.5 | 70.5 |
| | | S4 | | 71.5 | 68.1 | 75.5 |
| | | S5 | | 93.7 | 88.9 | 99.3 |
| | | S6 | | 56.3 | 49.4 | 62.9 |
| | | S7 | | 68 | 64.3 | 73.1 |
| 544 | 72h_+4°C | PC | Bacteriology | 89.8 | 82.5 | 94.5 |
| | | S1 | | 37.9 | 23.7 | 57.8 |
| | | S2 | | 0 | 0 | 0 |
| | | S3 | | 56.2 | 45.4 | 62.3 |
| | | S4 | | 83.7 | 76.1 | 90.8 |
| | | S5 | | 98.5 | 92.5 | 103.8 |
| | | S6 | | 0 | 0 | 0 |
| | | S7 | | 52.3 | 43.6 | 57.1 |
| | | PC | qPCR | 66.1 | 60.4 | 72.1 |
| | | S1 | | 59.6 | 57.1 | 61.5 |
| | | S2 | | 64.5 | 55.3 | 71.5 |
| | | S3 | | 65.8 | 62.5 | 71.1 |
| | | S4 | | 68.2 | 64.6 | 72.3 |
| | | S5 | | 93.5 | 90.9 | 95.4 |
| | | S6 | | 67.3 | 63.2 | 70.5 |
| | | S7 | | 59.7 | 51.8 | 65.3 |
| | | PC | Bacteriology | 34 | 23.6 | 44 |
| | | S1 | | 43.8 | 25.1 | 60.7 |
| | | S2 | | 0 | 0 | 0 |
| | | S3 | | 0 | 0 | 0 |
| | | S4 | | 31.5 | 14.8 | 49.7 |
| | | S5 | | 69 | 55.8 | 76 |

| | | | | | | |
|---|---|---|---|---|---|---|
| | 72h_-20°C | S6 | | 26.9 | 14.7 | 35.8 |
| | | S7 | | 0 | 0 | 0 |
| | | PC | qPCR | 67.8 | 61 | 74.9 |
| | | S1 | | 61.3 | 58.1 | 65.3 |
| | | S2 | | 62.1 | 52.4 | 70.7 |
| | | S3 | | 59.4 | 55.7 | 62.3 |
| | | S4 | | 70.6 | 64.7 | 78.7 |
| | | S5 | | 70.7 | 64.6 | 75.8 |
| | | S6 | | 66.6 | 62.5 | 69.4 |
| | | S7 | | 64.1 | 62.1 | 66.8 |
| | DT | PC | Bacteriology | 76.8 | 61.8 | 87.8 |
| | | S1 | | 55.9 | 35.5 | 72.4 |
| | | S2 | | 0 | 0 | 0 |
| | | S3 | | 78.8 | 68.1 | 88.3 |
| | | S4 | | 79.3 | 70.9 | 86.5 |
| | | S5 | | 94.9 | 89.5 | 100 |
| | | S6 | | 0 | 0 | 0 |
| | | S7 | | 46.6 | 19.6 | 62.3 |
| | | PC | qPCR | 74.8 | 71.4 | 78 |
| | | S1 | | 64.9 | 59.5 | 69.3 |
| | | S2 | | 64.8 | 63.9 | 65.3 |
| | | S3 | | 70.5 | 66.6 | 73.5 |
| | | S4 | | 71.1 | 68.9 | 73 |
| | | S5 | | 89.8 | 82.3 | 97.4 |
| | | S6 | | 53.3 | 49.5 | 57.4 |
| | | S7 | | 70.7 | 69.1 | 72.2 |
| | | PC | Bacteriology | 71.1 | 58.4 | 85.3 |
| | | S1 | | 19.9 | 0 | 50.1 |
| | | S2 | | 0 | 0 | 0 |
| | | S3 | | 24.6 | 0 | 44.4 |
| | | S4 | | 73.1 | 62 | 83 |
| | | S5 | | 92.6 | 87.9 | 98 |
| | | S6 | | 0 | 0 | 0 |

| | | | | | | |
|---|---|---|---|---|---|---|
| Thomsen | 72h_+4°C | S7 | | 52.8 | 41.9 | 65.6 |
| | | PC | qPCR | 72.2 | 67.3 | 76.7 |
| | | S1 | | 60.4 | 53.1 | 68.4 |
| | | S2 | | 70.2 | 68.2 | 71.7 |
| | | S3 | | 64.1 | 61.8 | 66.5 |
| | | S4 | | 68.7 | 64.3 | 72.8 |
| | | S5 | | 96.3 | 92.7 | 101.2 |
| | | S6 | | 60.2 | 59.3 | 61 |
| | | S7 | | 67.9 | 66.4 | 69.5 |
| | 72h_-20°C | PC | Bacteriology | 37.6 | 22.2 | 50 |
| | | S1 | | 0 | 0 | 0 |
| | | S2 | | 0 | 0 | 0 |
| | | S3 | | 0 | 0 | 0 |
| | | S4 | | 18.1 | 0 | 39.4 |
| | | S5 | | 83.8 | 78.4 | 89.4 |
| | | S6 | | 0 | 0 | 0 |
| | | S7 | | 0 | 0 | 0 |
| | | PC | qPCR | 68.1 | 65.3 | 71.2 |
| | | S1 | | 62.8 | 57.5 | 66.2 |
| | | S2 | | 66 | 63.2 | 68.6 |
| | | S3 | | 65 | 61.1 | 68.7 |
| | | S4 | | 66.7 | 61.9 | 71 |
| | | S5 | | 81.8 | 73.9 | 86.7 |
| | | S6 | | 61.1 | 59.8 | 62.6 |
| | | S7 | | 67.2 | 65.7 | 69.1 |
